# Delay QoS and MAC Aware Energy-Efficient Data-Aggregation Routing in Wireless Sensor Networks

**DOI:** 10.3390/s91007711

**Published:** 2009-09-28

**Authors:** Frank Yeong-Sung Lin, Hong-Hsu Yen, Shu-Ping Lin

**Affiliations:** 1 Department of Information Management, National Taiwan University, No. 1, Sec. 4, Roosevelt Rd., Taipei City 106, Taiwan; E-Mails: yslin@im.ntu.edu.tw (F.Y.S.L.); harry.splin@gmail.com (S.P.L.); 2 Department of Information Management, Shih Hsin University, No. 1, Lane 17, Sec. 1, Mu-Cha Rd., Taipei City 116, Taiwan

**Keywords:** delay QoS routing, MAC-aware data aggregation, energy efficient cross layer design, optimization, wireless sensor networks

## Abstract

By eliminating redundant data flows, data aggregation capabilities in wireless sensor networks could transmit less data to reduce the total energy consumption. However, additional data collisions incur extra data retransmissions. These data retransmissions not only increase the system energy consumption, but also increase link transmission delays. The decision of when and where to aggregate data depends on the trade-off between data aggregation and data retransmission. The challenges of this problem need to address the routing (layer 3) and the MAC layer retransmissions (layer 2) at the same time to identify energy-efficient data-aggregation routing assignments, and in the meantime to meet the delay QoS. In this paper, for the first time, we study this cross-layer design problem by using optimization-based heuristics. We first model this problem as a non-convex mathematical programming problem where the objective is to minimize the total energy consumption subject to the data aggregation tree and the delay QoS constraints. The objective function includes the energy in the transmission mode (data transmissions and data retransmissions) and the energy in the idle mode (to wait for data from downstream nodes in the data aggregation tree). The proposed solution approach is based on Lagrangean relaxation in conjunction with a number of optimization-based heuristics. From the computational experiments, it is shown that the proposed algorithm outperforms existing heuristics that do not take MAC layer retransmissions and the energy consumption in the idle mode into account.

## 1. Introduction

Wireless sensor networks (WSNs) have received attention increasingly in recent years. First, the sensor nodes can probe and collect environmental information, such as temperature, atmospheric pressure and irradiation by providing ubiquitous sensing, computing and communication capabilities. Second, thanks to the development of sensor node hardware technologies, the cost of sensor nodes has declined rapidly. This makes it possible to deploy large scale WSNs [1]. WSNs are similar to mobile *ad-hoc* networks (MANETs) in that they both involve multi-hop communications. However, there are two main differences. First, when an event occurs, multiple sensor nodes (denoted as data source nodes) around the event will transmit the sensed data back to one sensor node (denoted as the sink node). This is a *multipoint-to-point* mode, distinct from the communication between any pair of two nodes in MANETs. Second, since data are collected by multiple data source nodes and sent back to one sink node, it results in redundant data received by the relay node. Each relay node could collect and process these received data and transmit only one copy of the data back to the sink in such a way to save energy, if the data can be aggregated by nature. This kind of energy saving routing by redundant data elimination is known as the data aggregation routing. Besides redundant data elimination, other possible data aggregation function including maximum (MAX), minimum (MIN) and average (AVG) functions.

**Figure 1.**
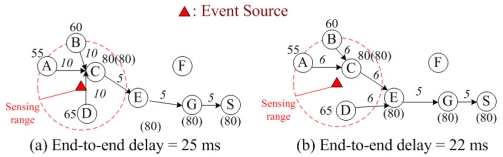
Data aggregation in MAX.

Figure 1 gives an illustrative example of returning the maximum temperature back to the sink node. The union of the routing paths from the data source nodes (i.e., *A, B, C, D*) back to the sink (i.e., *S*) constitutes the data aggregation tree. The number beside the node indicate the sensed temperature (in °F), and the number in parenthesis is the maximum temperature collected so far. Hence, node *E*, which is the relay node in the data aggregation tree, sends only the maximum temperature (80°F) from its children nodes (i.e., *C* and *D*) to node *G* to save energy. Note that the number (in mini-second) beside the link is the link delay (including the transmission delay for sending data and the latency from retransmission(s)), which will be described more clearly in Equation (4) in Section II.

Since each sensor node is powered by a battery and the exchange of batteries at the depleted sensor nodes is unlikely, data aggregation routing has been put forward as a particularly useful function for routing in terms of energy consumption in WSNs [2,3]. By data aggregation, redundant data could be eliminated. Based on this idea, energy-efficient routing is to encourage data aggregation as much as possible. However, the more flows are aggregated, the higher is the probability that the senders will experience data retransmissions [3].

In WSNs, any sensor node that is within another's interference range trying to transmit simultaneously would result in *collisions*. When collisions occur, *retransmissions* are required to ensure that the data be successfully received. These retransmissions result in additional energy consumption. Beside additional energy consumption, extra latency from retransmissions increases the link delay. Because of this extra latency for each link delay, the end-to-end delay from data source nodes back to the sink node will be increased.

For example, in Figure 1(a), the sensed data from three nodes (*A, B* and *D*) are aggregated at node *C* and then sends only one copy of data to node *E*. Because three nodes are aggregated at node *C*, the extra latency from retransmissions makes the link delay link *BC̅* (10 ms) larger than the link delay at Figure 1(b) (6 ms) where there are only two nodes aggregated at node *C*. Hence, from the delay QoS perspective, we should not perform too much data aggregation so as to meet the delay QoS requirement of the WSN. Besides the delay QoS requirement itself, the longer the end-to-end delay becomes, the larger the energy consumption will be caused for sensor nodes operating in the idle mode to wait for data from downstream nodes in the data aggregation tree. For example, in Figure 1(a), node *E* will operate in the idle mode for 15 ms to wait for data from nodes *A, B, C* and *D*. In Figure 1(b), node *E* will operate in the idle mode for 12 ms. Hence, in Figure 1(a), node *E* will consume more energy to operate in the idle mode than in Figure 1(b). The above example indicates that retransmissions introduce additional *energy consumption* and *delay*, which will jeopardize the advantages of data aggregations. An effective data aggregation algorithm should optimize the trade-off between energy saving from redundant data elimination and extra energy loss and additional delay from data retransmissions.

Basically, there are two operating stages (active stage and sleep stage) for sensor nodes in WSNs. In the sleep stage, a sensor node will turn off its transceiver so that there is no power consumption. Whereas, in the active stage, a sensor node could either transmit data or listen from other sensor nodes. To facilitate data communication, the sensor nodes in the data aggregation tree should be in the active stage, and all the nodes that are not included in the data aggregation tree (e.g., node *F* in Figure 1) are in the sleep stage to save energy. When a sensor node is transmitting data, it is in the transmission mode. When a sensor node is listening from other sensor nodes, it is in the idle mode. According to [4], the energy consumption for a sensor operating in the idle mode is slightly less than that of a sensor operating in the transmission mode. Hence, to capture the real energy consumption in WSNs, we should not only consider energy consumption in the transmission mode but also account for energy consumption in the idle mode.

When a sensor node in a data aggregation tree waits for data from the downstream nodes, this sensor node would operate in the idle mode during the waiting time (which is the maximum end-to-end delay from the farthest downstream node). For example, in Figure 1(a), node *G* would spend 20 (= 10 + 5 + 5) ms to operate in the idle mode to wait for data transmissions from its farthest downstream nodes (i.e., *A* and *B*) and then would spend 5 ms to operate in the transmission mode for data transmission. In this case, we observe that the power consumption in the idle mode is larger than that in the transmission mode. In addition, the additional retransmission latency that increases the end-to-end delay would make sensor nodes take longer time to operate in the idle mode, which in turn makes larger energy consumption. Therefore, the advantages of minimizing the end-to-end delay in WSNs include not only satisfying the delay QoS requirements of emergent events or real-time traffic but also minimizing the total energy consumption implicitly.

Intensive research has been conducted on data aggregation routing, but the important MAC layer retransmission issue as described above has been relatively seldom addressed. Krishnamachari [5] devises three interesting suboptimal aggregation heuristics, called Shortest Paths Tree (SPT), Center at Nearest Source (CNS), and Greedy Incremental Tree (GIT), respectively. In the SPT scheme, each data source node finds the shortest path back to the sink node. The CNS scheme selects one node that is the nearest to the sink node as the aggregation node and other data source nodes connect to this aggregation node by using respective shortest hop paths. And in the GIT scheme, initially the member(s) in the tree is only the sink node. Each data source finds a shortest hop path to this tree and the data sources with the minimum hop along with the intermediate nodes on this path are included in this tree. This process is repeated until all data source nodes are included in the tree. In [6], they propose centralized heuristic based on the Prim's minimal cost spanning tree algorithm to construct a data aggregation tree. This heuristic incorporates residual energy of sensor nodes into the Prim's algorithm in order to prolong the lifetime of sensor nodes. In [2], they propose a rigorous mixed integer mathematical formulation for the data aggregation routing problem and propose solution approaches based on Lagrangean relaxation.

Several works have addressed the MAC aware data aggregation routing problem in WSNs. In [3], they study the energy consumption tradeoffs between the data aggregation and retransmission in wireless sensor network by using the Carrier Sense Multiple Access with Collision Avoidance (CSMA/CA) MAC protocol. However, in this work, the energy consumption function does not consider the power consumption in idle mode that makes the proposed algorithm might not get the real energy efficient data aggregation tree. In addition, retransmission latency is not considered in [3] so that it does not guarantee the delay QoS. In [7], the authors propose the MAC Anycasting protocol to achieve spatial convergence, and the Randomized Waiting protocol to achieve the temporal convergence. These spatial convergence and temporal convergence properties maximize the advantages of data aggregation in Structure-free (i.e., no pre-constructed network structure) wireless sensor networks. In [8,9], the retransmission problem is circumvented by assigning different channels to the sensor nodes within each other's interference range. They devise interesting heuristics to tackle the data aggregation routing and channel assignment simultaneously.

Several works have addressed the latency issue for data aggregation in WSNs. In [10], they study the tradeoff between data aggregation and latency in WSNs. Data aggregation tree is constructed by using the earliest-first, randomized, nearest-first and weighted-randomized to identify the parent node to relay the data from the data source node back to the sink node. Then assign different time slot to every sensor node on data aggregation tree that has the same parent node without collision but with the penalty of large latency. In [11], they consider the latency issue in constructing a minimum energy data aggregation tree. A data aggregation tree is a balanced binary tree where initially the sink node finds the nearest two sensor node as its children, and each children node identify another two nearest node as its children node. This process is repeated until all data source nodes are included in this balanced data aggregation tree. After the data aggregation tree is determined, channel assignment is performed to minimize latency and transmission power. A similar idea is also shown in [12]. However, restricting the data aggregation tree to be the balanced binary tree might lead to long data aggregation tree that has larger end-to-end delay and energy consumption.

How to minimize the energy consumption of MAC-aware data aggregation routing in WSNs under end-to-end delay QoS constraints (denoted as the *E2EDAR* problem) is challenging. To the best of our knowledge, there is no existing literature addressing this E2EDAR problem. In this paper, for the first time, we address the interplay between the advantages of data aggregation (i.e., redundant data elimination) and the disadvantages of data aggregation (i.e., retransmission) to meet the delay QoS requirement and in the same time to achieve energy efficient routing which considers the energy consumption in transmission mode and in idle mode.

We propose an optimization-based heuristics to solve this E2EDAR problem. The problem is first formulated as an integer and non-convex mathematical programming problem where the objective function is to minimize total power consumption (includes data transmission power and the power consumption in idle time to wait data from downstream nodes) subject to data aggregation tree, transmission power coverage and end-to-end delay. Then Lagrangean relaxation scheme in conjunction with the optimization-based heuristics is proposed to solve this problem. From the computational experiments, the proposed solution approaches outperform the existing heuristics.

The remainder of this paper is organized as follows. In Section 2, a mathematical formulation of the E2EDAR is proposed. In Section 3, solution approaches based on Lagrangean relaxation are presented. In Section 4, heuristics are developed for calculating good primal feasible solution. In Section 5, computational results are reported. Finally, Section 6 concludes this paper.

## 2. Problem Formulation

We assume that each sensor node is equipped with a CSMA/CA compatible transceiver. In WSN, since there is no base station as the coordinator, the communication between sensor nodes is via ad-hoc mode. Hence, the sensor nodes will contend for the channel to transmit the data. We first examine the contention-based CSMA/CA protocol – Distributed Coordination Function (DCF), to derive the energy consumption function and delay function for the E2EDAR problem.

**Figure 2.**
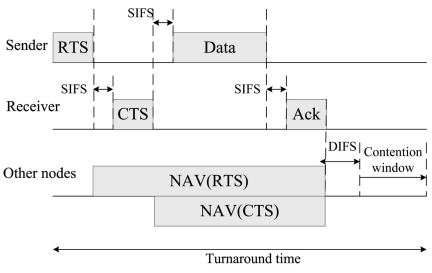
DCF mode in CSMA/CA protocol.

For the DCF in Figure 2, the turnaround time of each successful transmission is from RTS to the contention window. When a sender want to transmit data to receiver, it will first issue a RTS packet to ask the transmit permission from the receiver. If permission granted, the receiver will issue a CTS back to the sender, and then the data could be transmitted. After the data is successfully received, the receiver will transmit an ACK back to the sender. Because of this RTS and CTS mechanism, the hidden node problem will be circumvented. Other sensor nodes hear the RTS or CTS packet will not transmit to prevent collision in this NAV period. At the end of this round of transmission, all the sensor nodes will contend for the channel after a DIFS period. In order not to interrupt each round of transmission, the DIFS period is larger than the SIFS period.

Before we derive mathematical equations for the retransmission times and the end-to-end delay for data aggregation routing in WSNs, we first define notation in the following table.

The WSN is modeled as a graph in which sensors are represented as nodes and the arc connected two nodes indicates that one sensor is within the other's transmission radius. The definition of notation adopted in the formulation is listed below.

### 2.1. Notation in the Formulation

The notation used in the formulation is as follows.

Given parameters:
*N*The set of all sensor nodes*P_sq_*The set of all candidate paths by which data source node *s* can connect to the sink node *q**S*The set of all data source nodes*h*The maximum among all the shortest path distances from each of the data source nodes to the sink node*M*An arbitraryly large number*A*Maximum link delay*B*Maximum end-to-end delay*δ_p(n,k)_*The indicator function which is 1 if link (*n, k*) is on path *p* and 0 otherwise*d_nk_*Euclidean distance between node *n* and node *k**t_data_*Transmission time for transmitting a data packet*RTS*Transmission time for an RTS frame*CTS*Transmission time for a CTS frame*SIFS*Short inter-frame space time*DIFS*Distributed inter-frame space time*Θ*Maximum propagation delay for transmitting data packets*λ*Packet arrival rate*q*The sink node*R_n_*The set of all possible transmission radii that node *n* can adopt, which is a set of discrete elements*e_n_(r_n_)*Energy consumption function of node *n*, which is a function of node *n*'s transmission radius*E_idle_*Energy consumption per unit time when sensor nodes are operating in the idle mode*B̄*Average random back-off time*N̄*Average network allocation vector (NAV)*T*Maximum number of times for retransmissions

Decision variables:
*x_sp_*1 if data source node *s* uses path *p* to reach the sink node *q**y_(n,k)_*1 if link (*n, k*) is on the chosen data aggregation tree*r_n_*Transmission radius of node *n**l_(n,k)_*Data transmission delay from node *n* to node *k**m_n_*End-to-end delay from the farthest downstream node to node *n* on the data aggregation tree*z_nk_*1 if node *k* is covered within the transmission radius of node *n**c_nk_*Average number of retransmissions incurred at node *n* to transmit data to node *k*

Basically, the size of *P_sq_* will grow exponentially with number of sensor nodes (i.e., |*N*|.). So it is almost impossible to enumerate all possible paths at large network size. We will show in Section 3 that we do not need to enumerate all possible paths for *P_sq_*. The Lagrangean multipliers associated with decision variable *x_sp_* enable us to identify the shortest path for every data source node *s* by using the Dijkstra's shortest path algorithm. Hence, unlike applying a commercial optimization package (e.g., CPLEX) where all the values for *P_sq_* should be pre-specified, such explicit enumerations is unnecessary for the proposed Lagrangean relaxation scheme.

In order to better explain the objective function to be shown in the proposed mathematical formulation, we next provide a more detailed description on the network operation as follows. First, it is assumed that the network operates in a synchronous fashion, where in each “data collection cycle” all the nodes in the network cooperatively collect, aggregate and transmit a final result to the sink node. It is also assumed that a separate communication channel exists for, e.g., the sink node to initiate and control each data collection cycle. More precisely, during each data collection cycle, every data source node collects a measurement and sends it back to the sink node via the selected data aggregation tree. Once an intermediate node alone the data aggregation tree successfully receives data packets from all of its downstream nodes, the corresponding data aggregation process is performed and the intermediate result is transmitted upwards.

Based on the results in [3,13], we then conduct analyses on the average number of retransmissions in each data collection cycle for each sender node (either data source node or intermediate node) as follows. It is first assumed that each sensor node is equipped with a CSMA/CA compatible transceiver. For demonstration purposes, we next assume that each (re)transmission is an independent Bernoulli trial and that each sensor node generates packets (including retransmissions) with exponentially distributed interarrival times, where the mean is 1/λ. [This may correspond to the situation where (i) the transmission time for each new data packet from the cycle initiation time and (ii) the back-off time after a collision are both exponentially distributed with the mean 1/λ so as to avoid collisions.] Note that transmissions of data from a sender to a receiver are also assumed to be affected by the sensor nodes whose transmission radii cover the receiver due to interference. By considering the receiver side collisions in terms of communication radii of sensor nodes, the hidden terminal problem is also implicitly contemplated. We then derive the average number of retransmissions for node *n* to transmit data to node *k* (i.e., *c_nk_*) as follows:

(1) $$c_{\text{nk}}=\frac{1}{p_{\text{success}(n,k)}}=\frac{1}{e^{-\lambda (\text{RTS}+\text{SIFS}+2\theta )\sum_{j\in N}z_{\text{jk}}}}.$$

The meaning of the above retransmission function is the mean value of a geometrically distributed random variable where the successful transmission probability *p_success_*_(*n,k*)_ is that for a transmission from node *n* no data transmission is occurring at any other node whose transmission radius covers receiver node *k* within the interval of *RTS* + *SIFS* + *2θ*. Note that *p_success_*_(*n,k*)_ is in general an underestimation, where the maximum degree of interference during the entire data collection cycle is assumed. As a result, Equation (1) is considered a conservative/engineering approach to estimating the value of *c_nk_*.

The link transmission delay from node *n* to node *k* (i.e., *l_(n,k)_*) is calculated based on the analysis result in [13] and listed as follows:

(2) $$l_{\left(n,k\right)}=\frac{P_{\text{success}(n)}^{\text{DIFS}}(\text{RTS}+\text{SIFS}+\text{CTS}+\bar{B})+\text{DIFS}+\bar{N}}{P_{\text{success}(n)}^{\text{DIFS}}\cdot P_{\text{success}(n,k)}}-\bar{N}$$

where $P_{\text{success}(n)}^{\text{DIFS}}$ is defined as the probability of no data transmission occurring at any node whose transmission radius covers node *n* within *DIFS* interval when node *n* finishes NAV countdown and tries to start transmission. Under the assumptions of (i) the total number of retransmissions for a sensor node to successfully transmit a packet can be described by a geometric distribution and (ii) that each sensor node generates data packets governed by a Poisson process with mean rate λ, as in (1), the probability of successfully detecting no data transmission at node *n* within DIFS is:

(3) $$P_{\text{success}(n)}^{\text{DIFS}}=e^{-\lambda (\text{DIFS})\sum_{j\in N}z_{\text{jn}}}$$

By substituting (3) into (2), we get:

(4) $$l_{\left(n,k\right)}=\frac{\left(e^{-\lambda (\text{DIFS})\sum_{j\in N}z_{\text{jn}}}(\text{RTS}+\text{SIFS}+\text{CTS}+\bar{B})+\text{DIFS}+\bar{N}\right)}{e^{-\lambda (\text{DIFS})\sum_{j\in N}z_{\text{jn}}}e^{-\lambda (\text{RTS}+\text{SIFS}+2\theta )\sum_{j\in N}z_{\text{jk}}}}-\bar{N}$$

The denominator in Equation (4) calculates the average retransmission times of node *n* to transmit data to node *k* with consideration of the number of sensor nodes whose transmission radii cover the transmitter and receiver. The numerator in Equation (4) calculates the average turnaround time from the RTS to the contention time (see in Figure 2). Hence, by capturing the times of retransmissions at the transmitter and the receiver, the link transmission delay is equal to the average turnaround time multiplied by the times of retransmissions. The E2EDAR problem is then formulated as the following optimization problem (IP).

### 2.2. Mathematical Formulation for the E2EDAR Problem

#### Problem (P)

Objective function:

(5) $$Z_{\text{IP}}=\min\sum_{n\in N}\left({\text{t}}_{\text{data}}+(\text{RTS}\cdot \sum_{k\in N}c_{\text{nk}})\right)\cdot e_{n}(r_{n})+\sum_{n\in N}m_{n}\cdot E_{\text{idle}}$$

subject to:

(6) $$\begin{array}{cc} \sum_{p\in P_{\text{sq}}}x_{\text{sp}}\delta_{p(n,k)}\leq y_{\left(n,k\right)} & \;\forall s\in S,\forall n,k\in N \end{array}$$

(7) $$\sum_{n\in N}\sum_{k\in N}y_{\left(n,k\right)}\geq \max\{h,\left|\text{S}|\right\}$$

(8) $$\begin{array}{cc} \sum_{s\in S}\sum_{p\in P_{\text{sq}}}x_{\text{sp}}\delta_{p(n,k)}\leq \left|S\right|\cdot y_{\left(n,k\right)} & \;\forall n,k\in N \end{array}$$

(9) $$\begin{array}{cc} \sum_{p\in P_{\text{sq}}}x_{\text{sp}}=1 & \;\forall s\in S \end{array}$$

(10) $$\begin{array}{cc} \sum_{k\in N}y_{\left(n,k\right)}\leq 1 & \;\forall n\in N \end{array}$$

(11) $$\begin{array}{cc} \frac{r_{n}-d_{\text{nk}}}{M}\leq z_{\text{nk}} & \;\forall n,k\in N \end{array}$$

(12) $$\begin{array}{cc} z_{\text{nk}}d_{\text{nk}}\leq r_{n} & \;\forall n,k\in N \end{array}$$

(13) $$\begin{array}{cc} y_{\left(n,k\right)}\leq z_{\text{nk}} & \;\forall n,k\in N \end{array}$$

(14) $$\begin{array}{cc} (m_{k}+l_{\left(k,n\right)})-A(1-y_{\left(k,n\right)})\leq m_{n} & \;\forall n,k\in N \end{array}$$

(15) $$\begin{array}{cc} l_{\left(n,k\right)}=\frac{\left(e^{-\lambda (\text{DIFS})\sum_{j\in N}z_{\text{jn}}}(\text{RTS}+\text{SIFS}+\text{CTS}+\bar{B})+\text{DIFS}+\bar{N}\right)}{e^{-\lambda (\text{DIFS})\sum_{j\in N}z_{\text{jn}}}e^{-\lambda (\text{RTS}+\text{SIFS}+2\theta )\sum_{j\in N}z_{\text{jk}}}}-\bar{N} & \;\forall n,k\in N \end{array}$$

(16) $$\begin{array}{cc} c_{\text{nk}}\geq \frac{e^{-(1-y_{\left(n,k\right)})M}}{e^{-\lambda (\text{RTS}+\text{SIFS}+2\theta )\sum_{j\in N}z_{\text{jk}}}} & \;\forall n,k\in N \end{array}$$

(17) $$\begin{array}{cc} x_{\text{sp}}=0\;\text{or}\;1 & \;\forall s\in S,p\in P_{\text{sq}} \end{array}$$

(18) $$\begin{array}{cc} y_{\left(n,k\right)}=0\;\text{or}\;1 & \;\forall n,k\in N \end{array}$$

(19) $$\begin{array}{cc} z_{\text{nk}}=0\;\text{or}\;1 & \;\forall n,k\in N \end{array}$$

(20) $$\begin{array}{cc} r_{n}\in R_{n} & \;\forall n\in N \end{array}$$

(21) $$\begin{array}{cc} r_{n}\neq 0 & \;\forall n\in S \end{array}$$

(22) $$\begin{array}{cc} c_{\text{nk}}\in \{0,1,2,\ldots ,T\} & \;\forall n,k\in N \end{array}$$

(23) $$\begin{array}{cc} B\geq m_{n}\geq 0 & \;\forall n\in N \end{array}$$

(24) $$\begin{array}{cc} A\geq l_{\left(n,k\right)}\geq \text{RTS}+\text{SIFS}+\text{CTS}+\bar{B}+\text{DIFS} & \;\forall n,k\in N \end{array}$$

The first term in the objective function indicates the energy consumption from data packet transmission and RTS packet retransmissions. The second term in the objective function indicates the energy consumption in the idle period. Hence, the objective function of (IP) is to minimize data transmission power and the power consumption in idle time.

Constraint (6) requires that if path *p* is selected for source node *s* to reach the sink node *q*, the path must be on the tree. This constraint also enforces that if link (*n, k*) is on path *p* adopted by source node *s* to reach the sink node, then *y_(n,k)_* should be 1. Constraints (7) and (18) require that the total number of links on the aggregation tree is at least the maximum of *h* and the cardinality of *S*. Note that both *h* and |*S*| are legitimate lower bounds on the total number of links in an aggregation tree. Introducing Constraint (7) will significantly improve the solution quality. |*S*| and *h* could be calculated in advance. The left-hand-side of Constraint (8) calculates the number of paths, which are destined for the sink node and passed through link (*n, k*) on the data aggregation tree. The right-hand-side of Constraint (8) is at most |*S*|. When the union of the paths destined for the sink node does exist a cycle, and this cycle contains link *l*, then Constraint (8) would not be satisfied since there would be many paths passing through this link. Constraints (9) and (17) require that every data source adopt only one routing path destined for the sink node. Constraint (10) is an outgoing link constraint. All intermediate nodes on the aggregation tree should have only one outgoing link. Constraints (8), (9), (10), and (17) enforce that the union of all routing paths be a tree.

Constraints (11) and (12) are the number-of-neighbors constraints. If r_n_ ≥ d_kn_, *z_nk_* should be equal to 1 and 0 otherwise. Using *z_nk_* we can calculate the total number of sensor nodes whose transmission covers sensor node *k*, or the total number of sensor nodes covered by transmission radius of sensor node *n*. By jointly enforcing Constraints (11) and (12) we can model the relationship described above through decision variable *z_nk_*. Constraint (13) is a necessary constraint that relates decision variable *y_(n,k)_* to *z_nk_*. If *y_(n,k)_* equals to 1 then *z_nk_* also must be 1. Also note that Constraint (13) implicitly obligates that if sensor nodes were not on the data aggregation tree, they would not choose any link emanating from them as a link used by data aggregation tree, since this behavior would increase the cost of objective function (IP).

Constraint (14) calculates the maximum end-to-end delay for every sensor node *n* on the aggregation tree. Because of data aggregation, every sensor node on the data aggregation tree should wait all the sensed data from its downstream nodes. In other words, every sensor node on the data aggregation tree should wait for the data from the farthest children nodes. Due to the tree structure, there is a recursive relation to calculate the end-to-end delay for the sensor node. In Constraint (14), when link (*n, k*) is not on the tree (i.e., *y_(n,k)_* = 0), (*m_k_* + *l*_(_*_k,n_*_)_) − *A* ≤ *m_n_*. Since *A* is defined as the maximum link delay, it makes this constraint unbinding. On the other hand, when link (*n, k*) is on the tree (i.e., *y_(n,k)_* = 1), (*m_k_* + *l*_(*k,n*)_) ≤ *m_n_*. For example, in Fig. 1 (a), *m_A_* = *m_B_* = 0 ms and *l_(A, C)_* = 10 ms, so *m_C_* ≥ *m_A_* + *l_(A, C)_* = 10 ms. Then, *m_C_* = 10 ms and *l_(C, E)_* = 5 ms, so *m_E_* ≥ *m_C_* + *l_(C, E)_* = 15 ms. This recursive relation goes up along the tree until the sink node is reached. Then, *m_S_* ≥ *m_G_* + *l_(G, S)_* = 25 ms. Hence, by modeling this *recursive relation* in Constraint (14), we successfully capture the end-to-end delay for every sensor node on the data aggregation tree.

Constraint (15) calculates the link transmission delay. Due to two decision variable multiply each other at the numerator of Constraint (15), it is a non-convex programming problem and it is difficult to tackle it directly. In order to make Constraint (15) solvable, we approximate Constraint (15) by a simpler form and we guarantee that the approximation error is within five percent.


(25) $$\begin{array}{ll} l_{\left(n,k\right)} & =\frac{\left(e^{-\lambda (\text{DIFS})\sum_{j\in N}z_{\text{jn}}}(\text{RTS}+\text{SIFS}+\text{CTS}+\bar{B})+\text{DIFS}+\bar{N}\right)}{e^{-\lambda (\text{DIFS})\sum_{j\in N}z_{\text{jn}}}e^{-\lambda (\text{RTS}+\text{SIFS}+2\theta )\sum_{j\in N}z_{\text{jk}}}}-\bar{N}\\ & ≅\frac{e^{0.115+(0.017-\lambda (\text{DIFS}))\sum_{j\in N}z_{\text{jn}}}(\text{RTS}+\text{SIFS}+\text{CTS}+330)}{e^{-\lambda (\text{DIFS})\sum_{j\in N}z_{\text{jn}}}e^{-\lambda (\text{RTS}+\text{SIFS}+2\theta )\sum_{j\in N}z_{\text{jk}}}}\\ & =\frac{e^{0.115+0.017\sum_{j\in N}z_{\text{jn}}}(\text{RTS}+\text{SIFS}+\text{CTS}+330)}{e^{-\lambda (\text{RTS}+\text{SIFS}+2\theta )\sum_{j\in N}z_{\text{jk}}}} \end{array}$$

**Figure 3.**
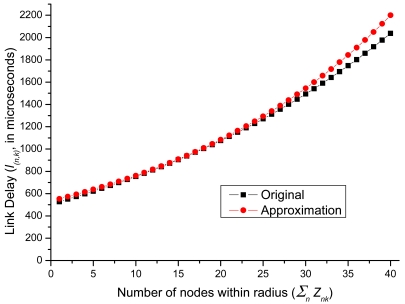
Link delay approximation function versus the original function.

In Constraint (25), the unit for 0.115, 0.017 and 330 is microsecond. In Figure 3, we show the difference between Constraint (15) and Constraint (25). For small $\sum_{n\in N}z_{\text{nk}}$ value, there is almost no difference between original function and approximation function. Even in large $\sum_{n\in N}z_{\text{nk}}$ value (e.g., = 40), there is only five percent difference.

In addition, the approximation function exceeds the original function. This guarantees that if the approximation function could satisfy the maximum end-to-end delay function from the data source node back to the sink, then the original function will also satisfy. This kind of overestimation is valid from the engineering perspective. We then take natural logarithm on both sides in order to make this function solvable:

(26) $$\ln(l_{\left(n,k\right)})=\ln\left(\text{RTS}+\text{SIFS}+\text{CTS}+330\right)+0.115+0.017\sum_{j\in N}z_{\text{jn}}+\lambda (\text{RTS}+\text{SIFS}+2\theta )\sum_{j\in N}z_{\text{jk}}$$

Constraint (16) is the calculation function of link retransmission times. For Constraint (16), we also take natural logarithm on both sides:

(27) $$\begin{array}{l} \ln(c_{\text{nk}})\geq \ln\left(\frac{e^{-(1-y_{\left(n,k\right)})M}}{e^{-\lambda (\text{RTS}+\text{SIFS}+2\theta )\sum_{j\in N}z_{\text{jk}}}}\right)\\ \Rightarrow \;\ln(c_{\text{nk}})\;\geq \;\lambda \;(\text{RTS}+\text{SIFS}+2\theta )\sum_{j\in N}z_{\text{jk}}-M+My_{\left(n,k\right)} \end{array}$$

Constraint (20) restricts that the set of possible transmission radii that node *n* can adopt is discrete and finite set. Constraint (21) enforces that each data source node should turn on its transmission radius. Hence, the transmission radius of each source node can not be 0. Constraint (22) is the bounding constraint of retransmission times. The bounding value is related to maximum end-to-end delay or can be obtain according to specification of standard. Constraint (23) is the lower bound and upper bound of maximum end-to-end delay. Constraint (24) enforces the lower bound of the link transmission delay of each link. Note that (*RTS*+*SIFS*+*CTS*+*B̄*+*DIFS*) is the turnaround time when there is no retransmission. Hence, it is the lower bound of the link transmission delay. Similarly, we could also approximate Constraint (24) as:

(28) $$A\geq l_{\left(n,k\right)}\geq e^{0.115}\cdot \left(\text{RTS}+\text{SIFS}+\text{CTS}+330\right)\;\forall n,k\in N$$

Problem (P) is a non-convex programming problem because a number of decision variables are coupled in product forms in Equations (5), (15) and (16). Besides such non-convex properties, integer constraints associated with several decision variables make (P) even more complicated. In problem (P), there are a total number of $\left(2\left|N\right|+4{\left|N\right|}^{2}+\sum_{s\in S}\left|P_{\text{sq}}\right|\right)$ decision variables. Besides an extremely large number of decision variables, problem (P) contains the data aggregation tree, transmission radius assignment, data retransmission and delay QoS routing problems. Since delay QoS constrained routing problems are well-known to be NP-hard [14], problem (P) is also NP-hard. We thus propose an optimization-based heuristic based on Lagrangean relaxation to tackle this problem.

## 3. Solution Approach–Lagrangean Relaxation

The algorithm development is based upon Lagrangean relaxation. In (IP), by introducing Lagrangean multiplier vectors *u^1^, u^2^, u^3^, u^4^, u^5^, u^6^, u^7^*, and *u^8^*, we dualize Constraints (6), (8), (11), (12), (13), (14), (26), and (27) to obtain the following Lagrangean relaxation problem (LR). Basically, the more constraints are relaxed, the looser duality gap between the solutions to the dual problem and the primal problem. Loose duality gap might indicate that the solution to the primal problem might be too far from the optimal solution. On the other hand, if too little constraints are relaxed, we might not be able to solve the Lagrangean dual problem optimally. Then the solution to the dual problem is not a legitimate lower bound of the primal problem. As will be shown in the following paragraph, by relaxing these eight constraints in (P), the resulting (LR) problem can be further decomposed into a number of mutually independent and easily solvable subproblems, which is essential for effectively solving the dual problem so as to obtain tight lower bounds.

### Problem (LR)


(LR) $$Z_{\text{LR}}=\min\sum_{n\in N}\left({\text{t}}_{\text{data}}+(\text{RTS}\cdot \sum_{k\in N}c_{\text{nk}})\right)\cdot e_{n}(r_{n})+\sum_{n\in N}m_{n}\cdot E_{\text{idle}}+\sum_{n\in N}\sum_{k\in N}\sum_{s\in S}u_{\text{nk}s}^{1}\left(\sum_{p\in P_{\text{sq}}}x_{\text{sp}}\delta_{p(n,k)}-y_{\left(n,k\right)}\right)+\sum_{n\in N}\sum_{k\in N}u_{\text{nk}}^{2}\left(\sum_{s\in S}\sum_{p\in P_{\text{sq}}}x_{\text{sp}}\delta_{p(n,k)}-|S|y_{\left(n,k\right)}\right)+\sum_{n\in N}\sum_{k\in N}u_{\text{nk}}^{3}(r_{n}-d_{\text{nk}}-Mz_{\text{nk}})+\sum_{n\in N}\sum_{k\in N}u_{\text{nk}}^{4}(z_{\text{nk}}d_{\text{nk}}-r_{n})+\sum_{n\in N}\sum_{k\in N}u_{\text{nk}}^{5}(y_{\left(n,k\right)}-z_{\text{nk}})+\sum_{n\in N}\sum_{k\in N}u_{kn}^{6}\left(m_{k}+l_{\left(k,n\right)}-A(1-y_{\left(k,n\right)})-m_{n}\right)+\sum_{n\in N}\sum_{k\in N}u_{\text{nk}}^{7}\left(\ln\left(\text{RTS}+\text{SIFS}+\text{CTS}+330\right)+0.115+0.017\sum_{j\in N}z_{\text{jn}}+\lambda (\text{RTS}+\text{SIFS}+2\theta )\sum_{j\in N}z_{\text{jk}}-\ln(l_{\left(n,k\right)})\right)+\sum_{n\in N}\sum_{k\in N}u_{\text{nk}}^{8}\left(\lambda (\text{RTS}+\text{SIFS}+2\theta )\sum_{j\in N}z_{\text{jk}}-M(1-y_{\left(n,k\right)})-\ln(c_{\text{nk}})\right)$$

subject to Constraints (7), (9), (10), (17), (18), (19), (20), (21), (22), (23) and (28).

(LR) is then decomposed into the following 6 independent subproblems.

Subproblem 1: for *m_n_*

(SUB1) $$\min\sum_{n\in N}\left(E_{\text{idle}}+\sum_{k\in N}u_{\text{nk}}^{6}-\sum_{k\in N}u_{kn}^{6}\right)m_{n}.(\text{SUB}1)$$

subject to (23).

Subproblem 2: for *y*_(*n,k*)_

(SUB2) $$\min\sum_{n\in N}\sum_{k\in N}\left(u_{\text{nk}}^{5}+u_{\text{nk}}^{6}A+u_{\text{nk}}^{8}M-u_{\text{nk}}^{2}|S\right|-\sum_{s\in S}u_{\text{nk}s}^{1})y_{\left(n,k\right)}$$

subject to (7), (10) and (18).

Subproblem 3: for *x_sp_*

(SUB3) $$\min\sum_{n\in N}\sum_{k\in N}\sum_{s\in S}\sum_{p\in P_{\text{sq}}}(u_{\text{nk}s}^{1}+u_{\text{nk}}^{2})x_{\text{sp}}\delta_{p(n,k)}$$

subject to (9) and (17).

Subproblem 4: for *r_n_* and *c_nk_*

(SUB4) $$\min\sum_{n\in N}e_{n}(r_{n})\cdot t_{\text{data}}+\text{RTS}\sum_{n\in N}\sum_{k\in N}e_{n}(r_{n})\cdot c_{\text{nk}}+\sum_{n\in N}\sum_{k\in N}(u_{\text{nk}}^{3}-u_{\text{nk}}^{4})r_{n}-\sum_{n\in N}\sum_{k\in N}u_{\text{nk}}^{8}\ln(c_{\text{nk}})$$

subject to (20), (21) and (22).

Subproblem 5: for *z_nk_*

(SUB5) $$\min\sum_{n\in N}\sum_{k\in N}\left(u_{\text{nk}}^{4}d_{\text{nk}}-u_{\text{nk}}^{3}M-u_{\text{nk}}^{5}\right)z_{\text{nk}}+\lambda (\text{RTS}+\text{CTS}+2\theta )+\sum_{n\in N}\sum_{k\in N}\sum_{j\in N}(u_{\text{nk}}^{7}+u_{\text{nk}}^{8})z_{\text{jk}}+0.017\sum_{n\in N}\sum_{k\in N}\sum_{j\in N}u_{\text{nk}}^{7}z_{jn}$$

subject to (19).

Subproblem 6: for *l*_(*n,k*)_

(SUB6) $$\min\sum_{n\in N}\sum_{k\in N}u_{\text{nk}}^{6}l_{\left(n,k\right)}-u_{\text{nk}}^{7}\ln(l_{\left(n,k\right)})$$

subject to (28).

(SUB1) can be further decomposed into |*N*| independent subproblems. For each node *n*∈ *N*,

(SUB1.1) $$\min\left(E_{\text{idle}}+\sum_{k\in N}u_{\text{nk}}^{6}-\sum_{k\in N}u_{kn}^{6}\right)m_{n}\left(\text{SUB1}.1\right)$$

subject to:

*B* ≥ *m_n_* ≥ 0.

When the coefficient of *m_n_* (i.e., $\left(E_{\text{idle}}+\sum_{k\in N}u_{\text{nk}}^{6}-\sum_{k\in N}u_{kn}^{6}\right)$) is positive, let *m_n_* = 0. When the coefficient of *C_l_* (i.e., $\left(E_{\text{idle}}+\sum_{k\in N}u_{\text{nk}}^{6}-\sum_{k\in N}u_{kn}^{6}\right)$) is negative, let *m_n_* = *B*. The computational complexity for this algorithm is *O*(1) for each node *n*.

Subproblem 2 is to determine decision variable *y*_(*n,k*)_.

The proposed algorithm to optimally solve (SUB2) is shown as follows.

**Step 1.** For every link (*n, k*), compute the coefficient $\left(u_{\text{nk}}^{5}+u_{\text{nk}}^{6}U+u_{\text{nk}}^{8}M-u_{\text{nk}}^{2}|S|-\sum_{s\in S}u_{\text{nk}}^{1}\right)$ for each *y_(n,k)_*. Then calculate the number of links whose coefficients are negative.

**Step 2.** For all outgoing links of node *n*, identify the link with the smallest coefficient. If the smallest coefficient is negative then set the corresponding *y_(n,k)_* to be 1 and the other outgoing links *y_(n,k)_* to be 0, otherwise set all outgoing link *y_(n,k)_* to be 0. Repeat Step 2 for all nodes.

**Step 3.** If the number of negative coefficient links (assume the number is *θ*) is smaller than max{*h_g_*, |*D_g_*|}, assign the corresponding *y_(n,k)_* = 1 for these negative coefficient links. Then sort those links that have positive coefficient in ascending order. Identify {max{*h_g_*, |*D_g_*|}–*θ*} number of smallest positive coefficient and let the corresponding *y_(n,k)_* = 1. Finally, let the other *y_(n,k)_* =0.

The computational complexity of the above algorithm is O(|*N*|^2^).

(SUB3) can be further decomposed into |*S*| independent shortest path problems with nonnegative arc weight whose value is $\left(u_{\text{nk}s}^{1}+u_{\text{nk}}^{2}\right)$. For each shortest path problem it can be effectively solve by the Dijkstra's algorithm. The computational complexity of the Dijkstra's algorithm is *O*(|*N*|^2^) for each data source node.

(SUB4) can be optimally solved by exhaustively searching all combinations of radius *r_n_* and *c_nk_*. The computational complexity of (SUB4) is therefore *O*(|*R_n_*| ×|*T*|) for each node *n*.

In (SUB5), if the corresponding coefficient $\left(u_{\text{nk}}^{4}d_{\text{nk}}-u_{\text{nk}}^{3}M-u_{\text{nk}}^{5}-2\lambda (\text{RTS}+\text{CTS}+2\theta )\sum_{j\in N}\left(u_{\text{jk}}^{7}+u_{\text{jk}}^{8}\right)+0.017\sum_{j\in N}u_{kj}^{7}\right)z_{\text{nk}}$ of link (*n, k*) is negative then set *z_nk_* to be 1, otherwise 0. The computational complexity of (SUB5) is *O*(1) for each link (*n*, *k*).

We can further decompose (SUB6) into |*N*|^2^ independent subproblems. For each link (*n, k*),

(SUB6.1) $$\min\;u_{\text{nk}}^{6}l_{\left(n,k\right)}-u_{\text{nk}}^{7}\;\ln(l_{\left(n,k\right)})$$

subject to:

*A* ≥ *l*_(*n,k*)_ *e*^0.115^·(*RTS* + *SIFS* + *CTS* + 330)

If $u_{\text{nk}}^{7}$ is negative then set *l*_(*n,k*)_ to be *e*^0.115^·(*RTS* + *SIFS* + *CTS* + 330). If $u_{\text{nk}}^{7}$ is positive then we can get the value of optimal value of *l*_(*n,k*)_ by the following procedure. Apply the first derivative with respect to *l*_(*n,k*)_ on the objective function of (SUB6.1) and let it be 0,

$$\begin{array}{l} \frac{\partial (u_{\text{nk}}^{6}l_{\left(n,k\right)}-u_{\text{nk}}^{7}\ln(l_{\left(n,k\right)}))}{\partial l_{\left(n,k\right)}}=u_{\text{nk}}^{6}-\frac{u_{\text{nk}}^{7}}{l_{\left(n,k\right)}}\\ \Rightarrow u_{\text{nk}}^{6}-\frac{u_{\text{nk}}^{7}}{l_{\left(n,k\right)}}=0\\ \Rightarrow l_{\left(n,k\right)}=\frac{u_{\text{nk}}^{7}}{u_{\text{nk}}^{6}}. \end{array}$$

Then calculate the second derivative with respect to *l*_(*n,k*)_ on the objective function of (SUB6.1).


$$\frac{\partial (u_{\text{nk}}^{6}-\frac{u_{\text{nk}}^{7}}{l_{\left(n,k\right)}})}{\partial l_{\left(n,k\right)}}=\frac{u_{\text{nk}}^{7}}{{\left(l_{\left(n,k\right)}\right)}^{2}}\geq 0,\;\text{since}\;u_{\text{nk}}^{7}\;\text{is positive}.$$

Since the second derivative is larger than or equal to zero, the objective function of (SUB6.1) is a convex function. Then the optimal value of *l*_(*n,k*)_ is either $\frac{u_{\text{nk}}^{7}}{u_{\text{nk}}^{6}}$ or the boundary points (i.e., *A* or *e*^0.115^ (*RTS* + *SIFS* + *CTS* + 330)) that leads to the minimum value of objective function of (SUB6.1). The computational complexity of (SUB6.1) is *O*(1) for each link (*n*, *k*).

According to the algorithms proposed above, we could effectively solve the Lagrangean relaxation problem optimally. Based on the weak Lagrangean duality theorem, *Z_D_*(*u^1^,u^2^,u^3^,u^4^,u^5^,u^6^,u^7^,u^8^*) is a lower bound on *Z_IP_*. We could calculate the tightest lower bound by using the Subgradient method [15]. Note that the solutions to the dual problem may not be feasible for the primal problem due to the fact several constraints are relaxed. In the sequel, we propose a heuristic for getting the primal feasible solution.

## 4. Obtaining Primal Feasible Solutions

The basic idea of getting primal feasible solution (denoted as *LGR-Primal*) is first to identify the energy efficient data aggregation and then adjust the routing path to meet the end-to-end delay constraint. We choose the routing decision variables *x_sp_* from (SUB3) at the beginning to get good primal feasible solutions. Once the routing path *x_sp_* for each data source node *s* is determined, all the other decision variables, e.g., *r_n_* and *y_nk_*, can be calculated and the total energy consumption of the data aggregation tree can be obtained.

We perform rerouting algorithm to decrease the maximum end-to-end delay of the routing path for each data source node. The steps of the rerouting heuristic are as follows:

1. Identify the path (denoted as *P*) that incurs the maximum end-to-end delay.
2. Investigate nodes located on *P* one by one. For each checked node (denoted as *n*), examine each node (denoted as *k*) within the transmission radius of *n*. If the maximum end-to-end delay of node *n* plus the link delay, *l*_(*n,k*)_, is smaller than the maximum end-to-end delay of node *k*, then reroute the outgoing link on the routing path of node *n* from the original routing link to the outgoing link (*n, k*). If no node *k* can be rerouted by *n*, then check the next node on *P* until the sink node is reached.
3. Update the decision variable *y*_(*n,k*)_ and recalculate the maximum end-to-end delay of the new routing path.
4. If no node on path *P* can be rerouted, then stop the heuristic; otherwise, go to Step 1.

However, the union of the routing paths of all data source node might not be a data aggregation tree because the tree constraints [i.e., Constraints (6) and (8)] are relaxed. We propose a drop heuristic to eliminate those links that form the cycle on the tree.

The algorithm for the drop heuristic is as follows:

1. Based on the solutions of (SUB3) we can get the set of decision variables, *x_sp_*, from which we can determine which link, *y_nk_*, is used on the routing path by source *s*. If *y_nk_* is 1, we set the arc weight of it corresponding link to be $\left(\frac{\sum_{s\in S}u_{\text{nk}s}^{1}}{\left|S\right|}+u_{\text{nk}}^{2}+u_{\text{nk}}^{6}+u_{\text{nk}}^{7}+u_{\text{nk}}^{8}\right)$; otherwise, we set the arc weight to be infinity.
2. According to the arc weight calculated in Step 1, we sort the links from small to large.
3. We sequentially remove the links from the largest arc weight to the smallest one, but we ignore the links with infinity costs. At the time when link, say link (*n, k*), is removed from the routing path, we need to check whether every source node still has a routing path to the sink node. If any source node is unable to reach the sink node after removing link (*n, k*), we restore link (*n, k*) onto the routing path. If every source still has a routing path to reach the sink node, we remove link (*n, k*) and investigate the next link with smaller arc weight until all the links used by the union of routing path *x_sp_* have been examined.

After executing the drop heuristic we get a data aggregation tree without any cycles. The computational complexity of this getting primal feasible heuristic (include rerouting and drop heuristic) is *O*(|*S*‖*N*|^3^). Note that the reasons that we choose $\left(\frac{\sum_{s\in S}u_{\text{nk}s}^{1}}{\left|S\right|}+u_{\text{nk}}^{2}+u_{\text{nk}}^{6}+u_{\text{nk}}^{7}+u_{\text{nk}}^{8}\right)$ as the arc weight for performing drop heuristics is because these multipliers provide useful information for selecting the links. The first and the second term (i.e., *u^1^* and *u^2^*) indicate the tree violation cost. According to the Lagrangean multiplier update procedure [15], *u^1^* and *u^2^* will be increased at the next iteration if the tree constraint (i.e., Constraints (1) and (2)) is violated. Similarly, *u^6^* and *u^7^* will be increased at the next iteration if the link delay constraint [i.e., Constraints (14) and (26)] is violated. Finally, *u^8^* will be increased at the next iteration if the retransmission times constraint [i.e., Constraint (27)] is violated. After several iterations, the links that violate these constraints will incur larger Lagrangean multipliers. Hence, by incorporating these multipliers into the arc weight setting, we try to drop the links that do not satisfy the tree, delay and retransmission constraints. In other words, by using the information from these Lagrangean multipliers, we get the energy efficient data aggregation tree with considering the *penalty cost from violating tree, delay and retransmission times constraints*.

In the following, we show the complete algorithm (denoted as LGR) to solve Problem (P). The computational complexity of the above LGR algorithm is *O*(|*N*|^4^) for each iteration.

**Algorithm 1:** The LGR Algorithm.
**Begin**
 *Input*: Network topology, data source nodes, sink node and end-to-end delay QoS requirements *Output:* Data aggregation tree *Initialize* Lagrangean multiplier vectors *u^i^*(0) = 0, ∀*i* = 1,2,…,8. UB = a very large positive number (e.g., *M*) and LB = a very small negative number (e.g., −*M*) //upper and lower bounds, respectively. *quiescence_age* = 0, and *step_size* = 2. **For**
*iteration* = 1 to *Max_Iteration_Number*, perform the following:  *Solve* Subproblem 1, Subproblem 2, Subproblem 3, Subproblem 4, Subproblem 5 and Subproblem 6.  *ComputeZ_LR_* in (LR).  **If**
*Z_LR_*(*u*) > *LB*   *LB* = *Z_LR_*(*u*) and *quiescence_age* = 0.  **Else**
*quiescence_age* = *quiescence_age* + 1.  **If**
*quiescence_age* = *Quiescence_Threshold*   *step_size* = *step_size/2* and *quiescence_age* = 0.  **Run** the *LGR-Primal* algorithm.  **Compute** the new upper bound *ub*.  **If**
*ub* < UB then UB = *ub*.  **Update** the *step_size*.  **Update** the Lagrangean multiplier vectors. **End For**
**End**


## 5. Computational Experiments

The proposed algorithms for solving E2EDAR problem are coded in C and run on a PC with an INTEL™ PIV-2G CPU. *Max_Iteration_Number* and *Improve_Threshold* were set to 2,000 and 30, respectively. The step size coefficient, *δ*, is initialized as 2 and is halved when the objective function value of the dual problem is not improved in the number of *Improve_Threshold* iterations.

We assume that a sensor network operates in periodic mode where, the sensor nodes periodically report information to the sink node. The network topology comprises *N* = 150 sensor nodes randomly placed in a 1 × 1 square unit area. The most top left node is selected as the sink node such that we could have a data aggregation tree with larger depth. The cost of the energy consumption function, *e_n_*(*r_n_*) (in milliwatts), is defined as the {square of (100) × (Euclidean distance) × (energy consumption per millisecond when the sensor node is transmitting data)} (i.e., signal attenuation constant *α* = 2). The energy consumption values for a sensor node in the transmitting mode and in the idle mode (i.e., *E_idle_*) are all based on the experimental sensor node, MEDUSA, which is a low power sensor node developed by UCLA [4]. The set of all possible transmission radii of a sensor node *n* (i.e., *R_n_*) is a discrete set and it is configured to begin from 0 to the maximum transmission radius with 0.01 step size. To evaluate the solution quality of our proposed algorithm, we implement four existing algorithms for comparison. The GIT and the CNS algorithms are proposed in [5] and the third algorithm, CCA, is proposed in [16]. The forth algorithm, LGRMAC, is proposed in [3].

**Figure 4.**
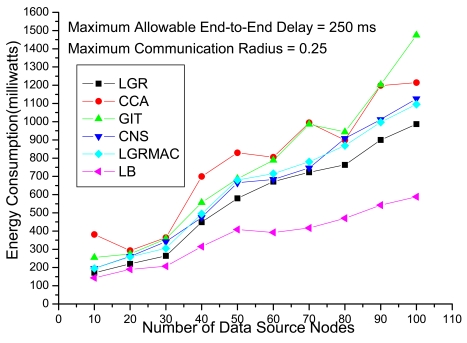
Performance comparison with respect to traffic loads.

Figure 4 shows the total energy consumption with respect to the number of data source nodes under loose delay QoS requirements. Recall that in the objective function of (IP) (i.e., Equation (5)), the total energy function consists of energy consumption in transmissions (including data transmissions and retransmissions) and energy consumption in the idle period. For a data aggregation tree with a large delay, even though the delay constraint is not violated under loose delay QoS requirements, large energy consumption will still be incurred due to long idle time. The other heuristics (CCA, GIT, CNS and LGRMAC), that do not take the idle time energy consumption into account, may become less effective in calculating energy-efficient data-aggregation trees. On the other hand, the LGR algorithm that considers the penalty from delay and retransmissions can get the most energy-efficient data aggregation tree.

In Figure 4, we also show the Lagrangean lower bounds (denote as LBs), which are theoretical lower bound on the optimal objective function value of the primal problem. The primal feasible solutions (i.e., LGR) are upper bound on the E2EDAR problem. Based on the weak Lagrangean duality theorem, the optimal solution must fall within the upper bound and the lower bound [15]. The gap between LB and LGR is defined as (LGR – LB)/(LB) × 100%, which is an upper bound on how far LGR is from an optimal solution. It is observed that the gap is increasing with respect to the number of data source nodes, which is about 15% when the number of data source nodes is small and about 70% when the number of data source nodes becomes large.

**Figure 5.**
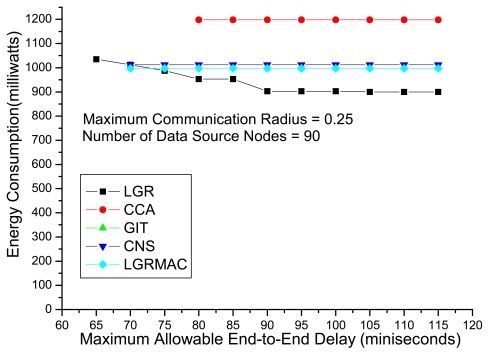
Performance comparison with respect to maximum end-to-end delays (i.e., *B*).

Figure 5 shows the energy consumption with respect to the maximum end-to-end delay (i.e., *B*). We observe that the CCA algorithm could obtain a feasible solution when *B* = 80 ms, and *B* = 70 ms for the CNS and the LGRMAC algorithms. The GIT algorithm could not identify any feasible solution even when *B* = 115 ms. For the LGR algorithm, a feasible solution can be found even when *B* = 65 ms. Besides LGR algorithm could locate feasible solution under stringent delay QoS requirements, the energy consumption is lower than the other heuristics under all maximum end-to-end delay. On the other hand, the CCA, the CNS and the LGRMAC heuristics always obtain the same data aggregation tree (i.e., energy consumption) regardless the value of the maximum allowable end-to-end delay. This is because these heuristics do not take the retransmission delay into consideration and only consider the energy consumption in the transmission mode. This kind of data aggregation design philosophy not only do not leverage on the loose delay QoS to minimize the total energy consumption but also is not applicable under stringent delay QoS requirements.

**Figure 6.**
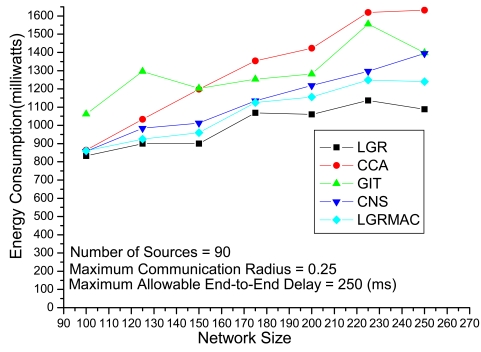
Performance comparison with respect to network sizes.

Figure 6 depicts the experiments evaluating the solution quality of different algorithms under different network sizes with 90 fixed sources and a loose delay constraint. Intuitively, when in large network size, there would be more data retransmission due to severe collisions. In other words, the solution approach should be more careful not to incur larger energy loss from data retransmission in large network size. Algorithms that do not address the retransmission will suffer from the extra energy loss from data retransmission. According to Figure 6, we observe that the LGR algorithm outperforms the other heuristics, especially for large network sizes. This indicates that the LGR algorithm optimizes the trade-off between advantages from data aggregations and disadvantages from data retransmissions.

We summarize the improvement ratio of the LGR algorithm over the other 4 heuristics in Table 1. The improvement ratio is defined as (other approach – LGR)/(LGR) × 100% to show the solution quality. In Table 1, the improvement ratio of LGR over LGRMAC, CCA, CNS and GIT is up to 17%, 123%, 30% and 49%, respectively.

## 6. Conclusions and Discussion

Data aggregation could decrease redundant data transmissions so as to minimize the total transmission energy. However, data aggregation also increases the collision probabilities so as to increase the system energy consumption and link delays from data retransmissions. Optimizing the trade-off between data aggregation and retransmission in terms of energy consumption and delay is an interesting and challenging issue in WSNs. In this paper, for the first time, we model the E2EDAR problem as an optimization problem, where the objective function is to minimize the total (including transmission time and idle time) energy consumption subject to delay QoS, retransmission and data aggregation tree constraints. The proposed solution approach is based on Lagrangean relaxation for calculating an energy-efficient data aggregation tree that considers routing assignment, transmission radius assignment, data retransmission, and maximum end-to-end delay constraints. Note that the values of Lagrangean multipliers reflect the violation cost for the corresponding relaxed constraints. Hence they could provide useful information to get primal feasible solutions. According to the computational experiments, the LGR algorithm is superior to the other heuristics under all tested cases. More precisely, the LGR algorithm outperforms the LGRMAC, CCA, CNS, and GIT heuristics by 17%, 123%, 30% and 49%, respectively. In addition, the LGR algorithm could not only obtain feasible data aggregation trees than the other heuristics under stringent delay QoS constraints but also identify more energy efficient data aggregation trees under loose ones.

Besides the objective considered in this paper to construct an energy-efficient data-aggregation tree so as to meet the delay QoS requirements, it is also an important design issue for an MSN to maximize its life time. Although the proposed model and algorithm may inherently tend to prolong the lifetime of a WSN, nevertheless, to specifically address the aforementioned issue of MSN lifetime, particularly taking into consideration of the effects of sensors' residual energy, the following mechanisms may be adopted directly based upon the proposed algorithm:

1. The proposed algorithm may be re-executed periodically or on an event-driven basis. When the residual energy of a node is below a certain level, this node is reserved for future use unless it is absolutely necessary.
2. Also execute the proposed algorithm periodically or on an event-driven basis, where the energy consumption function *e_n_*(*r_n_*) for each node *n* may be multiplied by a factor at each decision stage to reflect a penalty caused by short of energy.

## Figures and Tables

**Table 1.** Performance comparison between LGR and the other three heuristics.

**Heuristic**	**Network Load (no. of data source nodes)**	**End-to-end Delay Constraint (*B*)**	**Network Size**

LGRMAC	17%	11%	10%
CCA	123%	33%	50%
CNS	30%	12%	28%
GIT	49%	NA*	44%

* The GIT algorithm does not identify feasible solutions.
